# DHA exhibits synergistic therapeutic efficacy with cisplatin to induce ferroptosis in pancreatic ductal adenocarcinoma via modulation of iron metabolism

**DOI:** 10.1038/s41419-021-03996-y

**Published:** 2021-07-15

**Authors:** Jing Du, Xu Wang, Yanchun Li, Xueying Ren, Yi Zhou, Wanye Hu, Chaoting Zhou, Qiangan Jing, Chen Yang, Luyang Wang, Huanjuan Li, Lijuan Fang, Yonglie Zhou, Xiangmin Tong, Ying Wang

**Affiliations:** 1 Laboratory Medicine Center, Department of Laboratory Medicine, Zhejiang Provincial People’s Hospital, Affiliated People’s Hospital, Hangzhou Medical College, Hangzhou, Zhejiang 310014 China; 2 Department of Central Laboratory, Affiliated Hangzhou first people’s Hospital, Zhejiang University School of Medicine, Hangzhou, Zhejiang 310006 China; 3 Bengbu Medical College, Bengbu, Anhui 233000 China; 4 Zhejiang University of Technology, Hangzhou, Zhejiang 310014 China; 5 Department of Laboratory Medicine, Hangzhou Ninth People’s Hospital, Hangzhou, Zhejiang 310014 China; 6 Phase I Clinical Research Center, Zhejiang Provincial People’s Hospital, Affiliated People’s Hospital, Hangzhou Medical College, Hangzhou, Zhejiang 310014 China

**Keywords:** Chemotherapy, Preclinical research

## Abstract

Pancreatic ductal adenocarcinoma (PDAC) is an extremely lethal cancer with limited treatment options. Cisplatin (DDP) is used as a mainstay of chemotherapeutic agents in combination with other drugs or radiotherapy for PDAC therapy. However, DDP exhibits severe side-effects that can lead to discontinuation of therapy, and the acquired drug resistance of tumor cells presents serious clinical obstacles. Therefore, it is imperative to develop a more effective and less toxic therapeutic strategy. We and others have previously discovered that dihydroartemisinin (DHA) represents a safe and promising therapeutic agent to preferentially induce cancer cell ferroptosis. In the present study, we find that DHA could intensively strengthen the cytotoxicity of DDP and significantly reduce its effective concentrations both in vitro and in vivo. Combination of DHA and DDP synergistically inhibits the proliferation and induces DNA damage of PDAC cells. Mechanically, the combinative treatment impairs mitochondrial homeostasis, characterized by destroyed mitochondrial morphology, decreased respiratory capacity, reduced ATP production, and accumulated mitochondria-derived ROS. Further studies show that ferroptosis contributes to the cytotoxic effects in PDAC cells under the challenge of DHA and DDP, together with catastrophic accumulation of free iron and unrestricted lipid peroxidation. Moreover, pharmacologic depleting of the free iron reservoir or reconstituted expression of FTH contributes to the tolerance of DHA/DDP-induced ferroptosis, while iron addition accelerates the ferroptotic cell death. In summary, these results provide experimental evidence that DHA acts synergistically with DDP and renders PDAC cells vulnerable to ferroptosis, which may act as a promising therapeutic strategy.

## Introduction

Pancreatic ductal adenocarcinoma (PDAC) is the most common type of pancreatic cancer which is an extremely lethal cancer with poor prognosis and high recurrence rate. PDAC often harbors the universal mutations in the proto-oncogene *K-RAS* (>90% prevalence in pancreatic cancer), which persistently accelerates and activates various oncogenic events (e.g., uncontrolled proliferation, sustained angiogenesis, metastasis, or invasion), thus leading to metabolic reprogramming and resistance to cell death [1, 2]. Most patients with pancreatic cancer were diagnosed at a late stage even with distant metastasis and died within several months. Although the diagnosis and treatment of pancreatic cancer have achieved great progress, the outcomes of patients are still not satisfactory, especially in those patients with *K-Ras* oncogenic mutant [3]. The survival benefits of standard chemotherapies are still limited with a median survival of fewer than 6 months [4], the 5-year survival rate for pancreatic cancer patients remains less than 10% [5]. Therefore, it is imperative to develop more effective and less toxic therapies that sensitize cancer cells to chemotherapy agents. Ferroptosis, a new mode of regulated cell death (RCD), is more prone to occur in *Ras* mutant cancer cells, which might open up a new strategy to solve this problem [6].

Cisplatin (DDP), an effective platinum-based chemotherapeutic agent, has been used to treat various types of solid tumors, including lung, breast, esophageal, ovarian, and pancreatic cancers [7, 8]. The inhibition of proliferation through DNA damage in rapidly dividing cells is the main anticancer mechanism of DDP. Other mechanisms of DDP-induced cytotoxicity are involved in impairing glycolysis, mitochondrial dysfunction, and accumulation of reactive oxygen species (ROS) [9]. However, DDP exhibits severe side-effects that can lead to discontinuation of therapy and acquired drug resistance, which may contribute to the treatment failure in pancreatic cancer [10]. Currently, the depletion of glutathione caused by DDP and the inactivation of glutathione peroxidase were found to play a vital role in its underlying mechanism [11, 12], suggesting that combination chemotherapy based on ferroptosis could be developed to strengthen the therapeutic effect and reduce the cytotoxicity of DDP.

Due to the high cost and long time needed to develop a new therapeutic drug, drug-repurposing can be a faster and less costly alternative approach to the development of new drugs [13]. Artemisinin (ART), an extract derived from the Chinese plant *Artemisia annua* [14], has been widely used as an antimalarial drug with high safety and efficiency. In addition to the advantages of antimalarial effect, ART and its derivatives, including artesunate, dihydroartemisinin (DHA), and artemether, have been reported to exhibit strong antiviral, antibacterial, and anticancer activities [15, 16]. DHA, the main active metabolite of ART, has been reported to be capable of inhibiting cancer growth in lymphocytic leukemia, breast cancer, cervical cancer, and liver cancer with its active endoperoxide bridge (R-O-O-R0) [17]. Recent studies have demonstrated that DHA induced cell death via suppressing JAK2/STAT3, JNK1/2, NF-κB, and p38 MAPK signaling pathways [18–20]. Our previous study revealed that DHA represents a promising therapeutic agent to preferentially target AML cells and induced ferroptosis through degradation of ferritin [21]. Considering this fact, we are wondering whether DHA could act synergistic effects with cisplatin through inducing pancreatic cancer cells ferroptosis.

In this work, we examined the synergistic effect of DHA and DDP, and found DHA could intensively strengthen the cytotoxicity effect of DDP by inhibition of cell proliferation and migration, impairing mitochondrial functions, thus eventually resulting in ferroptotic cell death. Mechanistically, we identified that the synergistic effect of DHA and DDP is associated with the degradation of GPX4 and the accumulation of free iron. Pharmacological or genetic blocking of the free iron accumulation could largely block the synergistic effect of DHA and DDP both in vitro and in vivo. Of note, we firstly present the detailed experiments to uncover the mechanism of the synergistic cytotoxicity of DHA and DDP in treating pancreatic cancer cells and present a prospective therapeutic strategy through ferroptosis.

## Methods

### Cell lines

The pancreatic cancers cell lines PANC1 and SW1990 were preserved and passaged by our laboratory and maintained in DMEM medium (Hyclone, Logan, UT, USA) containing 10% fetal bovine serum (Gibco, Grand Island, USA), supplementary with 100 U/mL penicillin and 100 μg/mL streptomycin at 37 °C in an atmosphere of 5% CO_2_.

### Reagents and antibodies

The antibodies to Ferritin Heavy Chain (ab65080), GPX4 (ab125066), to NRF2 (ab62352), to MDA (ab27642), to GAPDH (ab181602), and to γ-H2AX (ab81299) were obtained from Abcam (Cambridge, MA). The antibody to TFR (sc-32272) was obtained from Santa Cruz (Dallas, USA). The antibodies to IRP2 (23829-1-AP), to FSP1 (20886-1-AP), and to DRP1 (12186-1-AP) were obtained from Proteintech (Wuhan, China). The antibody to XCT (12691) was obtained from Cell Signaling Technology (Danvers, MA). The antibody to 4-HNE (MAB3249) was obtained from Novus Biologicals (Littleton, CO, USA). The antibody to NCOA4 (203674-T08) was obtained from Sino Biological (Beijing, China). CCK-8 Assay Kit was obtained from Meilunbio (Dalian, China), Ferrostatin-1, Z-VAD-FMK, and Necrosuifonamide were obtained from Selleck Chemicals (Houston, TX). C11-BODIPY (581/591), MitoTracker were obtained from Thermo Fisher Scientific (Waltham, MA). Desferrioxamine (DFO) and 2ʹ,7ʹ-dichlorofluore scindiacetate (DCF-DA) were purchased from Sigma-Aldrich (St. Louis, USA).

### Cell viability assay

The cytotoxicity of DHA and DDP in pancreatic cancer cells was evaluated by the CCK8 assay Kit. PANC1 and SW1990 cells were seeded in a 96-well culture plate (NEST Biotechnology) at a density of 1.5 × 10^4^ cells per well in 100 μL medium overnight. Then, different concentrations of DHA and DDP (20–45 µM for PANC1 or 40–120 µM for SW1990) were added and incubated for 24 h, respectively. For the rescue assay, DHA and DDP were co-treated with a range of pharmacological inhibitors of specific cell death pathways including DFO (100 µM), ferrostatin-1 (fer-1, 1 µM), GSH (0.5 mM), Necrosulfonamide (0.5 µM), Z-VAD-FMK (5 µM), XL019 (2.5 µM), JSH-23 (10 µM), SB203580 (10 µM), SP600125 (10 µM), and SCH772984 (10 µM) for 24 h. After treatment, 10 μL CCK-8 was added to each well, and incubation continued for 2 h at 37 °C. The absorbance was measured at 450 nm on the microplate reader. The effect of the drug combination was determined based on the combination index (CI) computed by CompuSyn software. CI values below 1 indicate synergistic effects, whereas CI values above 1 suggest an antagonism between the drugs, CI values in the 0.9–1.10 range mainly indicate additive effects.

### Live/Dead staining assay

PANC-1 cells were seeded in 6-well plates (2 × 10^5^/well) and cultured for 24 h. DHA (40 µM) and DDP (40 µM) were added to the indicated culture plate wells for 24 h. Then, cells were harvested and subjected to Calcein-AM/PI staining kit (Beyotime, Shanghai, China) at 37 °C for 15 min. Nuclei were counterstained with DAPI (10 μg/mL) for 5 min. Fluorescence microscope (Nikon) was employed to take photos; PI stained for the dead cells, and Calcein AM stained for the live cells. Treated cells were also incubating with 5 μL Annexin V-FITC and 10 μL PI for 5 min at room temperature in the dark according to the manufacturer’s instructions (MultiSciences, Hangzhou, China). Single-cell suspensions were subjected to flow cytometry (ACEA NovoCyte, USA) and the percentage of Live/Dead cells was quantified.

### Colony formation assays

PANC-1 and SW1990 cells (1 × 10^3^/well) were seeded in 12-well plates and cultured for 24 h. The medium was then replaced with complete culture medium containing DHA (5 µM for PANC1 or 10 µM for SW1990) and DDP (5 µM for PANC1 or 10 µM for SW1990), or a combination of the two compounds for an additional 7 days. The colonies were fixed with 4% paraformaldehyde after washing with PBS, stained with 0.1% crystal violet for 30 min, and then the colonies were imaged and counted by ImageJ software.

### Edu incorporation assay

The 5-ethynyl-2-deoxyuridine (EdU) incorporation assay was carried out to test the cell proliferation capacity of PANC1 cells treated with mono DHA and DDP or the combination according to the manufacturer’s instructions (Beyotime, Shanghai, China). Images were viewed and captured under a confocal microscope.

### Cell cycle analysis

Cell cycle distribution was examined by flow cytometry. PANC1 cells were cultured in the serum deprivation medium for 24 h to synchronize the cell cycle. Following the corresponding treatments, cell cycle distribution was detected by Cell Cycle Staining Kit (MultiSciences, Hangzhou, China) according to the manufacturer’s protocol. Briefly, PANC1 cells were harvested and fixed with 70% cold ethanol. After fixation, the PI staining solution with RNase A was added and incubated in dark at 37 °C for 30 min. Stained samples were tested by flow cytometry (ACEA NovoCyte, USA).

### Confocal microscopy assay

The cells were seeded in a chamber confocal dish. After treatment for 12 h, cells were incubated with C11-BODIPY(5 μM) or MitoTracker (100 nM) or RPA (4 μM) in the dark for 30 min, nuclei were counterstained with DAPI (10 μg/mL). Then cells were washed three times with PBS and photographed under a confocal microscope (Leica, Germany).

### Oxygen consumption rate (OCR) determination

The oxygen consumption rate (OCR) was determined by a Seahorse XFe24 Bioanalyzer (Seahorse Bioscience). On the first day, PANC1 cells were placed in a XFe24 Seahorse Cell Culture Microplate (Seahorse Bioscience) at a density of 3 × 10^4^ cells/well. Meanwhile, the XFe24 sensor cartridges were hydrated. On the following day, the cells were treated for 8 h with DHA and DDP using aforementioned concentrations, then the cell media was changed to basic Seahorse DMEM containing 10 mM glucose, 2 mM glutamine, and 1 mM sodium pyruvate, plates were then incubated at 37 °C in a CO_2_-free incubator for 1 h before placing in the analyzer; OCR was measured with sequential injection of 1.5 μM oligomycin, 1.5 μM carbonyl cyanide-chlorophenylhydrazone (CCCP), and 0.5 μM Antimycin A/Rotenone.

### Determination of ROS production

Cellular ROS level and mitochondria-derived superoxide were measured using 2ʹ,7ʹ-dichlorofluore scindiacetate (DCF-DA) and mitoSOX probe, respectively. Following indicated treatments, PANC1 and SW1990 cells were washed with PBS and stained with DCF-DA (4 μM) or mitoSOX (3 μM) in the dark at 37 °C for 30 min. Cells were then washed with PBS, and the fluorescence intensity was measured by flow cytometry.

### Immunofluorescence

Cells were grown on glass coverslips placed in the 24-well culture plate. After designated treatments, cells were followed by fixation with 4% formaldehyde and permeabilization with 1% Triton X-100. Fixed cells were washed with PBS and blocked in 5% BSA, and then incubated with primary antibodies (Rabbit anti-MDA, Mouse anti-4HNE, Mouse anti-TFR, Rabbit anti-γ-H2AX) at 4 °C overnight. After washing twice with PBS, Alexa Fluor 488-labeled anti-rabbit IgG and Alexa Fluor 594-labeled anti-mouse antibody (Thermo, Waltham, MA) were added on the glass coverslips for 1 h, nucleus was stained with DAPI. Subsequently, monolayer cell images were observed and recorded under a laser scanning confocal microscope.

### Lentiviral packaging and transduction

Full-length FTH was ordered from Sino Biological (Beijing, China) and amplified by PCR (AP131-11, TransGen Biotech), then subcloned into pLVX-IRES-Neo lentivirus vector (Takara, Dalian, China) by ClonFast Seamless Cloning kit (Obio, Nanjing, China). The recombinant lentiviral plasmid was verified by sequencing. The recombinant lentiviral plasmid was co-transfected with pMD2.G, pSPAX2 into 293T cells to produce recombinant lentiviral. After 72 h of transfection, the supernatants were collected, centrifuged, and filtered through a 0.4 μm filter. To generate stably transfected cell lines, PANC1 cells (8 × 10^4^) were seeded in 24-well plates. After 12 h, the culture medium was removed, and cells were transfected with the corresponding lentivirus. After 2 days of transfection, 1 mg/mL G418 was added for selection for 7 days. Then the stable cells were maintained in 0.2 mg/mL G418. The transduction efficiency was evaluated by western blot analysis.

### Western blotting

Following treatment, the cells were harvested and lysed in RIPA buffer on ice for 10 min. The protein concentration was quantified using BCA Protein Assay Kit. Subsequently, equal amounts of protein were separated by 10% SDS-PAGE and transferred to PVDF membranes. The membranes were blocked with 5% non-fat milk for 1 h at room temperature and incubated with the primary antibodies at 4 °C overnight. After being washed three times with TBST, the membranes were incubated with the secondary antibodies at room temperature for 1 h and washed again. The blots were visualized using an ECL-Plus chemiluminescence detection kit.

### In vivo tumor model

Six-week-old female BALB/c nude were purchased from Jiangsu Jizui Yao Kang Biological Technology Co. LTD. PANC1 cells were harvested and washed with cold PBS three times, then the cell density was adjusted to 2 × 10^7^/mL in cold DMEM. All animals were injected subcutaneously with 2 × 10^6^ cells into the right dorsal flank. Once the tumors reached 80–100 mm^3^, mice were randomly allocated into groups and treated with vehicle, DHA (50 mg/kg), DDP (5 mg/kg), DHA plus DDP, and the combination plus DFO (100 mg/kg), respectively.

### Hematoxylin-eosin (H&E) staining

Tumors dissected from mice were fixed in 4% paraformaldehyde, embedded into paraffin. The embedded samples were cut to 4 μm thickness, deparaffinized and stained with H&E (Sigma) routinely. Stained sections were viewed and photographed under a microscope.

### Immunohistochemistry (IHC)

Tissues were fixed with 4% paraformaldehyde and embedded in paraffin. The embedded block tissues were cut into 4 μm sections and followed by dewaxing, dehydration, and antigen retrieval. After being washed with PBS three times, the slides were treated with 3% hydrogen peroxide for 15 min for blocking the endogenous peroxidase activity, then blocked with 5% BSA for 15 min at room temperature. Subsequently, anti-TFR antibody (1:50) and anti-Ki67 antibody (1:100) were used for incubation at 4 °C overnight. The streptavidin peroxidase method was used for signal detection and then stained by diaminobenzidine (DAB) and counterstained with hematoxylin, the sections were observed and photographed under the light microscope.

### Statistical analysis

All statistical calculations were performed using GraphPad Prism (version 7.0). Results are represented as mean ± SD. The differences between the two groups were performed by the Student’s *t*-test. Comparisons among multiple groups were analyzed by one-way ANOVA. Statistical significance was defined at ^★^*P* < 0.05, ^★★^*P* < 0.01, compared to the corresponding control.

## Results

### DHA exerts a synergistic effect with DDP, and significantly reduces its effective concentrations

**Fig. 1:**
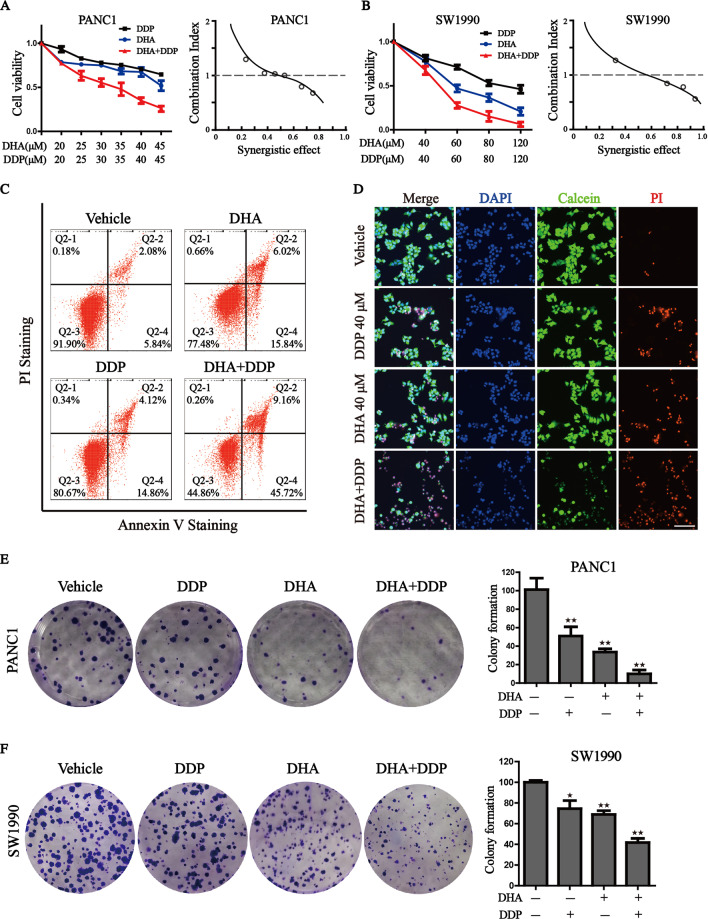
Synergistic antitumor effect of DHA in combination with DDP. **A**, **B** Pancreatic cancer cells were subjected to different concentrations of DHA and DDP treatment for 24 h and cell viability was detected by CCK-8 assay. Combination index (CI) values were calculated by Compusyn software. **C** PANC1 cells were subjected to 40 µM DHA or/and DDP treatment for 24 h and followed by Annexin V-FITC/PI assay. **D** PANC1 cells were pretreated with 40 µM DHA or/and DDP for 24 h and subjected to the Calcein-AM/PI staining (Calcein AM: live cells, PI: dead cells). Scale bar: 100 µm. **E**, **F** The colony formation assay of PANC1 and SW1990 cells were performed under mono or combination treatment of DHA (5 µM for PANC1 or 10 µM for SW1990) and DDP (5 µM for PANC1 or 10 µM for SW1990) for 7 days. The representative images and the corresponding quantitative histograms from three independent experiments are shown (values represented mean ± SD. ^★^*P* < 0.05, ^★★^*P* < 0.01 versus control).

Sustained DDP treatments for pancreatic cancer cells are ineffective and may lead to high drug resistance. Considering the previous study mentioning that DHA exerts a high capacity in cancer treatment [21], we wondered whether DHA combination with DDP could effectively optimize its antitumor activity. Firstly, we treated human pancreatic cancer cell lines (PANC1 and SW1990) with DHA and DDP, and tested the cytotoxicity against the two *K-Ras* mutant PDAC cell lines through CCK-8 assay. The combination index was calculated by Compusyn software. The results showed that DHA and DDP each inhibited the growth of pancreatic cancer cells in a dose-dependent manner. In addition, they exerted a synergistic effect with the increase of the concentrations, suggesting that DHA may be used as novel drug candidates to enhance the efficacy of DDP in pancreatic cancer treatment (Fig. 1A, B). The synergetic effect was obtained when 40 µM DDP was combined with 40 µM DHA for PANC1, and 60 µM DDP was combined with 60 µM DHA for SW1990. We utilized the Annexin V-FITC/PI assay and Calcein-AM/PI staining to further clarify the synergetic effect of DHA and DDP. Both of the experiments demonstrated that the combination of DHA and DDP synergistically increased the amounts of dead cells and decreased the amounts of viable cells (Fig. 1C, D). In addition, we examined the combined effect of DHA and DDP on the capacity of long-term cell viability by the clonogenic survival assay in PANC1 and SW1990 cells. As a result, clone clusters of the two pancreatic cancer cell lines exposed to the combinative treatment were less and smaller compared with either agent alone after culture for 7 days (Fig. 1E, F). Collectively, these results suggested that DHA remarkably strengthens the anticancer effect of DDP.

### Combination of DHA and DDP inhibits the proliferation and induces DNA damage in pancreatic cancer cells

**Fig. 2:**
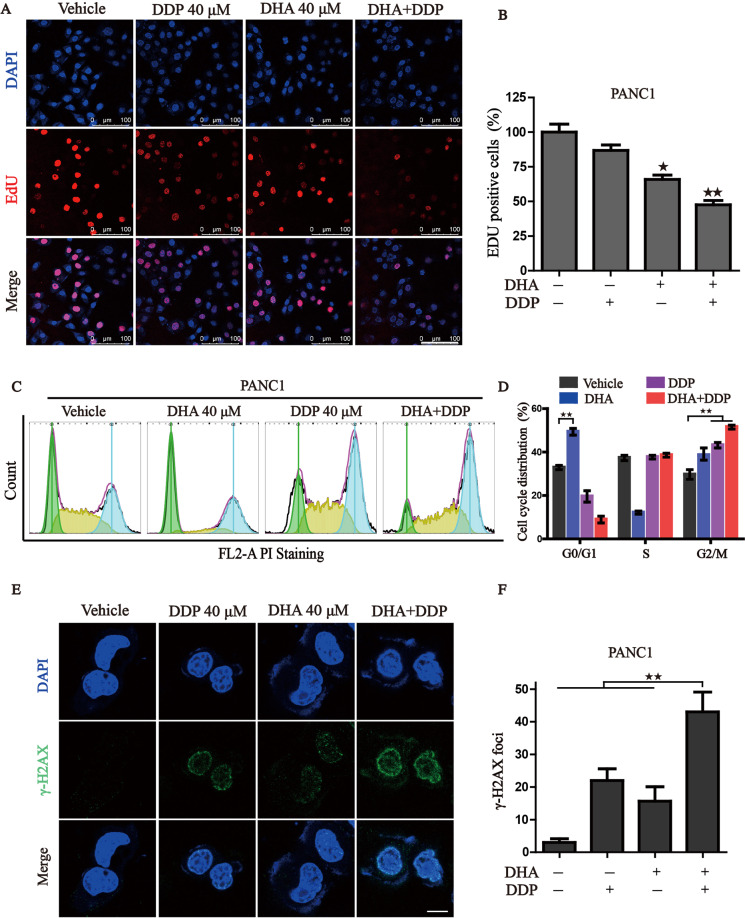
Combinative treatment of DHA and DDP inhibits proliferation and induces DNA damage in pancreatic cancer cells. **A**, **B** Cell proliferation of PANC1 was detected by 5-ethynyl-2′-deoxyuridine (EdU) incorporation assay under mono or combination treatment of DHA and DDP for 12 h. Representative images and statistical histograms are shown. Scale bar: 100 µm. **C**, **D** PANC1 cells were treated with mono or combination of DHA and DDP for 12 h, cell cycle distribution was determined by flow cytometry. **E**, **F** PANC1 cells were treated with mono or combination of DHA and DDP for 12 h and stained with γ-H2AX antibody. DAPI was used for nucleus staining. Images were acquired with confocal laser scanning microscopy. Quantification of γ-H2AX immunofluorescence was shown on the right. Scale bar: 10 µm. ^★^*P* < 0.05, ^★★^*P* < 0.01 versus control.

Next, we wonder whether the combination treatment could suppress pancreatic cancer cell proliferation. As expected, either DHA or DDP treatment moderately inhibited EdU incorporation into cell nuclei, while DHA co-administration with DDP caused a remarkable reduction in the percentage of EdU^+^ cells, suggesting that DHA in combination with DDP significantly disrupted pancreatic cancer growth (Fig. 2A, B). We have previously demonstrated that DHA suppresses the proliferation of AML cells by arresting the cell cycle [21]. To further investigate the mechanism of growth inhibition, we performed flow cytometry to analyze the cell cycle distribution. As shown in Fig. 2C, D, significant changes in the distribution of cell cycle were found in PANC1 cells under the treatment of DHA and DDP, with a dramatically increased proportion of G2/M phase and decreased proportion of G0/G1. As the primary anticancer mechanism of DDP is an interaction with DNA to form intrastrand crosslink adducts, resulting in elevated DNA damage and cell cycle arrest. We further detected the expression of γ-H2AX, a marker in the early stage of DNA damage, by immunofluorescence. Results showed that DHA sensitizes PANC1 cells to DDP-induced DNA damage, characterized by the dramatically increased amounts of γ-H2AX foci (Fig. 2E, F). Taken together, our data support a synergistic role of DHA and DDP in repressing the proliferation and inducing DNA damage of pancreatic cancer cells.

### Combined treatment of DHA and DDP synergistically impairs mitochondrial homeostasis

**Fig. 3:**
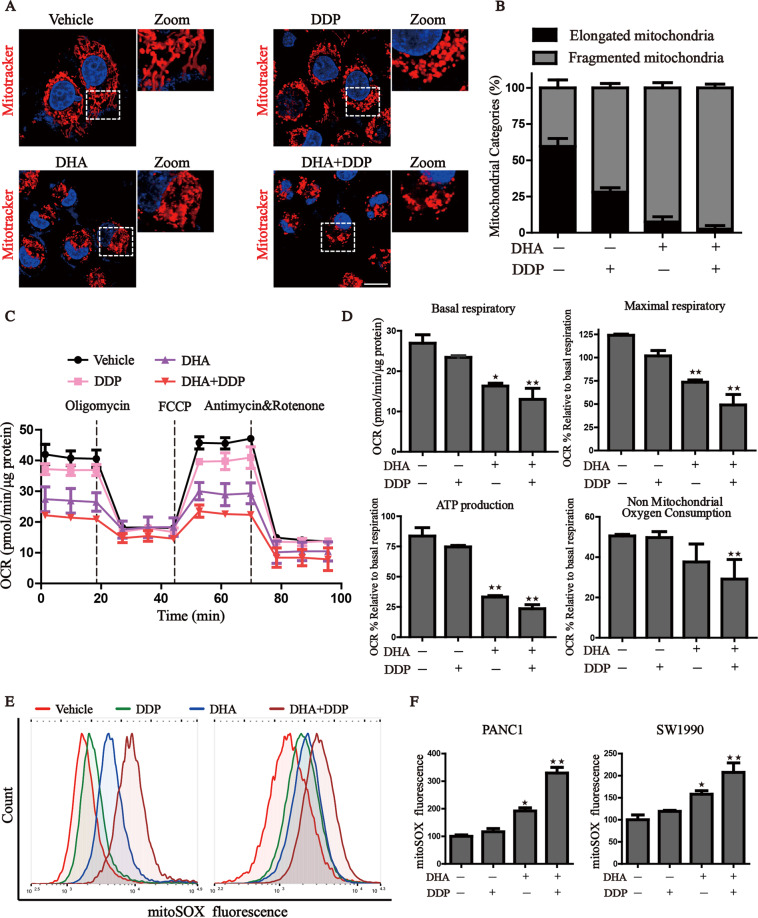
DHA co-treatment with DDP synergistically impairs mitochondrial homeostasis. **A**, **B** Observation of the changes in mitochondrial morphology. The treated PANC1 cells were stained with MitoTracker probe (100 nM) and DAPI (10 μg/mL) and then photographed by confocal laser microscope. Scale bars: 10 μm. **C**, **D** Mitochondrial oxygen consumption rate (OCR) was carried out with a Seahorse analyzer after the sequential addition of oligomycin, FCCP, and Antimycin A/ Rotenone. The OCR values of maximal respiration, ATP and non-mitochondrial oxygen consumption were normalized to the basal respiration. **E**, **F** Flow cytometry was performed to measure mitochondrial ROS by mitoSOX probe (3 μM) (values represented mean ± SD. ^★^*P* < 0.05, ^★★^*P* < 0.01 versus control).

Given the links between DHA and mitochondrial dysfunction, we then tested whether DHA combined with DDP would amplify mitochondrial dysfunction in pancreatic cancer cells. We first focused on mitochondrial morphology, which is an upstream event of mitochondrial dysfunction. Following the administration of DHA/DDP, the mitochondria became disorganized, smaller, and networks collapsed around the perinuclear region (Fig. 3A), which is more serious than the mono-treatment group. Statistical analysis revealed that administration of DHA/DDP decreased the proportion of elongated mitochondria, and induced a pronounced increase of fragmented mitochondria (Fig. 3B). Once mitochondrial dysfunction occurs, mitochondrial oxidative phosphorylation process may be affected. We then explored the mitochondrial respiratory capacity through Seahorse XFe24 Extracellular Flux Analyzer (Fig. 3C). The OCR, an indicator of mitochondrial respiration, represented that DHA acted synergistically with DDP to suppress basal respiratory, maximal respiratory, non-mitochondrial oxygen consumption and thus lead to the reduction of ATP production (Fig. 3D). We also detected mitochondrial ROS production, which originates from the electron leakage of the electron transport chain and results in cellular oxidative stress. The results determined by MitoSOX probe staining validated that DHA reinforced the mitochondrial ROS production under the challenge of DDP (Fig. 3E, F). These results demonstrate that the combinative treatment destroys mitochondrial homeostasis and facilitates the accumulation of mitochondria-derived ROS.

### Ferroptosis contributes to the cytotoxic effects in PDAC cells under the treatment with DHA and DDP

**Fig. 4:**
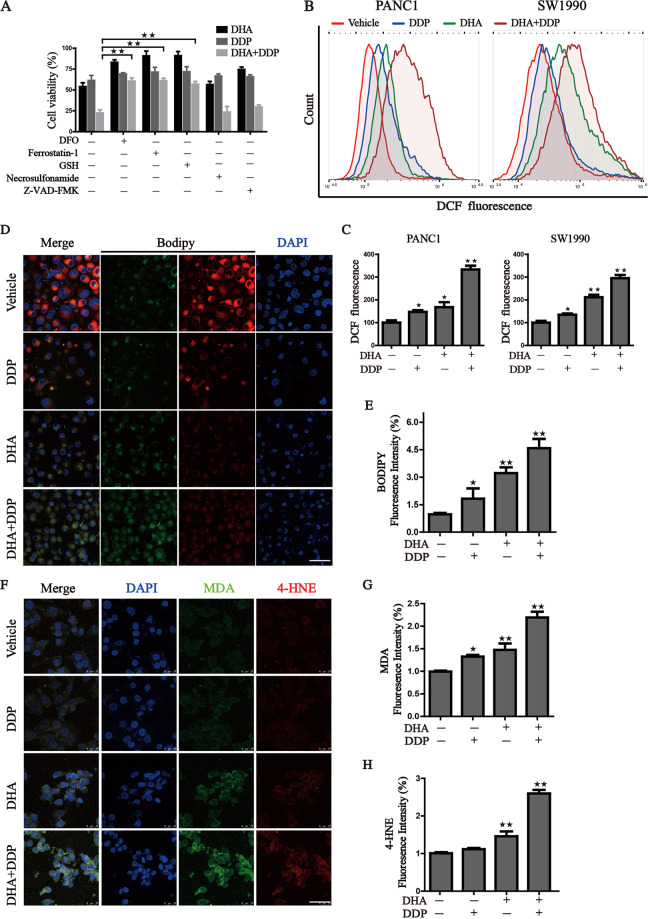
Ferroptosis contributed to the cytotoxic effects in PDAC cells under the treatment with DHA and DDP. **A** Cell viability was detected by CCK8 assay under treatment of DHA and DDP (45 μM) in the presence or absence of several cell death inhibitors. **B**, **C** Flow cytometry was performed to measure cellular ROS, and quantitation of fluorescence intensities was shown. **D**, **E** Representative images of BODIPY staining were photographed by confocal laser microscope with the designed treatment. Scale bars: 50 μm. Statistical results of the fluorescence intensities were shown on the right. **F**–**H** Immunofluorescence staining of MDA and 4-HNE were captured by confocal laser microscope with the designed treatment. Scale bars: 50 μm. Statistical results of the fluorescence intensities were shown on the right (values represented mean ± SD. ^★^*P* < 0.05, ^★★^*P* < 0.01 versus control).

To further characterize the basis of cell death induced by DHA/DDP, we treated PDAC cells with mono- or combination treatment of DHA and DDP in the absence or presence of several cell death inhibitors, including deferoxamine (DFO, iron-chelating agent), ferrostatin-1 (fer-1, ferroptosis inhibitor), Z-VAD-FMK (apoptosis inhibitor), Necrosulfonamide (necroptosis inhibitor) and GSH. Iron chelating agent, ferroptosis inhibitor and GSH significantly alleviate the decline of cell viability challenge by DHA and DDP (Fig. 4A). Western blot results also verified that pyroptosis and necroptosis may not participate in the cell death induced by DHA and DPP (Fig. S1). Recent studies have demonstrated that DHA induced cell death via suppressing JAK2/STAT3, NF-κB, and MAPK signaling pathways. Then, XL019 (JAK2 inhibitor), JSH-23 (NF-κB inhibitor), SB203580 (p38 inhibitor), SP600125 (JNK inhibitor), SCH772984 (ERK inhibitor) were incubated with DHA and DPP. Inhibition of NF-κB, JAK2, and ERK could further strengthen the cytotoxicity effect of DHA/DPP (Fig. S2), demonstrating that not these pathways but ferroptosis contribute to the main cytotoxic effect induced by DHA and DDP. The generation of lipid peroxides and accumulation of free iron are two major hallmarks of ferroptosis. Therefore, we quantified the oxidative stress damage by the following assays: (i) cellular total ROS generation indicated by DCF-DA probe (Fig.4B, C), (ii) lipid ROS measured by BODIPY C11 fluoroprobe (Fig. 4D, E), and (iii) secondary products of lipid peroxidation detected by immunofluorescence staining of MDA and 4-HNE (Fig. 4F–H). These results indicated that combinative treatment triggered an evident increase in cellular ROS production and lipid peroxide generation.

**Fig. 5:**
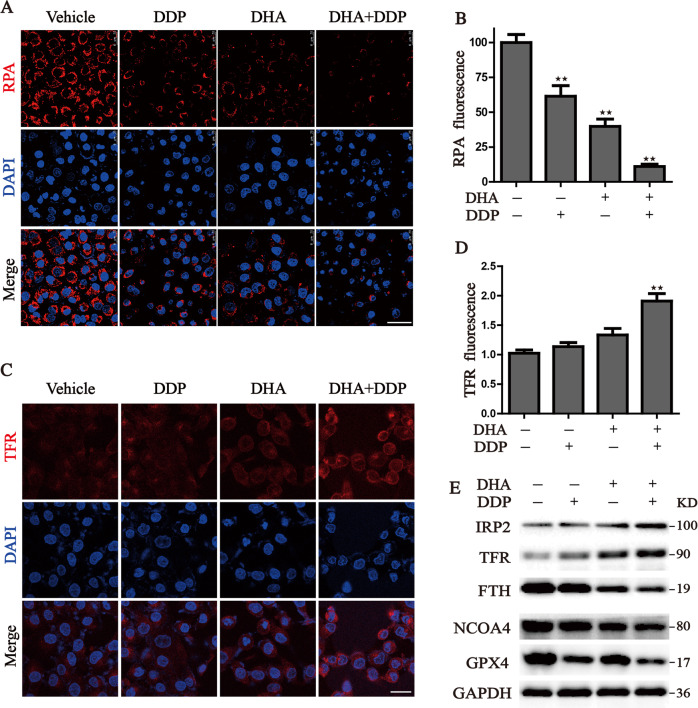
Iron accumulation in pancreatic cancer cells induced by the combination treatment of DHA and DDP. **A**, **B** Intracellular Fe^2+^ was measured by the staining of RPA and photographed by the confocal microscope after indicated treatment. Scale bars: 50 μm. **C**, **D** Detection of cell surface TFR levels by immunofluorescence staining of PANC1 cells after indicated treatment. **E** The expression of iron metabolism proteins in DHA- and DDP-treated cells, which was determined by western blot assay (values represented mean ± SD. ^★^*P* < 0.05, ^★★^*P* < 0.01 versus control).

To check whether the increase of lipid peroxidation is due to the increase of free iron pool. We examined the changes in iron metabolism in PDAC cells treated with DDP and DHA. We first utilized the selective yield fluorescence probe RPA to monitor the free iron level. The RPA staining images captured by confocal microscopy reflected that DDP combined with DHA significantly resulted in the accumulation of labile iron level manifested by the decreased fluorescence of RPA (Fig. 5A, B). TFR (Transferrin receptor) which imports iron from the extracellular environment into cells, is a specific ferroptosis marker and can be used to label cells undergoing ferroptosis [22]. We then detected the TFR expression via immunofluorescence staining (Fig. 5C, D). Consistent with RPA staining, accumulated TFR is located on the cell membrane under treatment with DHA and DDP, leading to the dramatic accumulation of unchelatable iron. We further investigated the expression of key proteins associated with iron homeostasis in pancreatic cancer cells. Similar results were observed that proteins related to iron metabolisms, such as IRP2, FTH, and FTL were regulated by DHA, and the iron-starvation effect was further enhanced under DDP co-treatment in PANC1 cells. Besides, DDP synergistic with DHA to induce the degradation of GPX4 and NCOA4, an autophagy cargo receptor that binds ferritin for degradation and releases free iron (Fig. 5E). Collectively, our results demonstrate that combinative treatment triggered an evident accumulation of free iron pool, resulting in the initiation of ferroptosis.

### Pharmacologic depletion of the free iron reservoir attenuates the DHA/DDP-induced ferroptosis

**Fig. 6:**
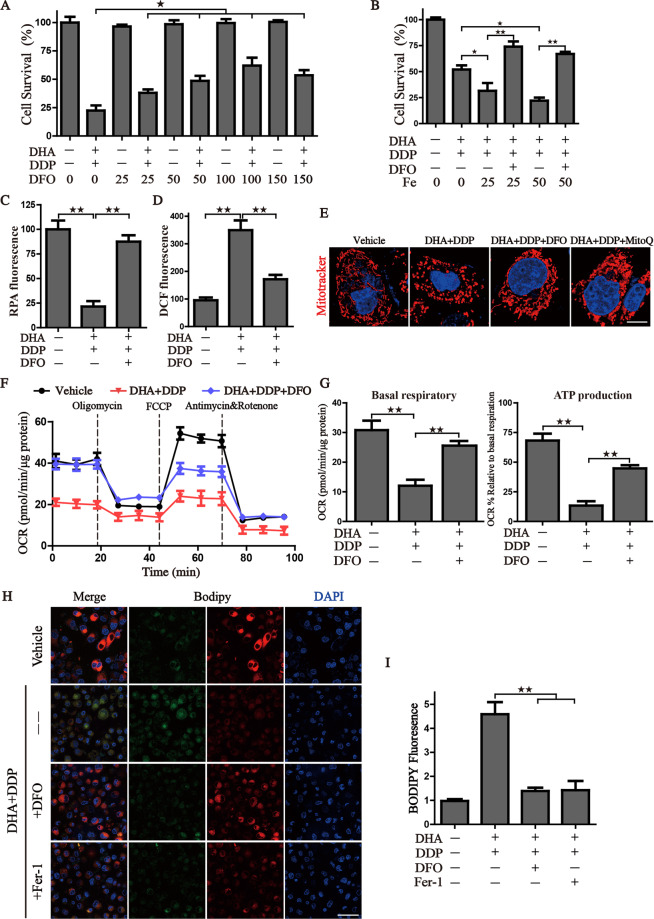
Pharmacologic depleting of the free iron reservoir attenuates the DHA/DDP-induced ferroptosis. **A** PANC1 cells were treated with DHA/DDP (45 μM) in the presence or absence of DFO. **B** PANC1 cells were treated with DHA/DDP (30 μM) in the presence or absence of DFO and Fe^2+^, cell survival was detected by CCK8. **C**, **D** After indicated treatment, PANC1 cells were loaded with RPA or DCF-DA probe for 30 min, and fluorescence intensities were detected by microplate spectrophotometer and normalized to the corresponding cell number. **E** MitoTracker Red labeled pancreatic cancer cells were subjected to the confocal microscope for observing the changes of mitochondrial morphology after the indicated treatment. Scale bars: 10 μm. **F**, **G** OCR of PANC1 cells was carried out with a Seahorse analyzer after the addition of oligomycin, FCCP, and Antimycin A/Rotenone. The basal respiration and ATP production were calculated on the right. **H**, **I** To assess lipid ROS production, pancreatic cancer cells were treated with DHA and DDP with or without DFO, fer-1. Treated cells were loaded with BODIPY C11 probe for 30 min followed by confocal laser microscope. Scale bars: 50 μm. Statistical results of the fluorescence intensities were shown on the right (values represented mean ± SD. ^★^*P* < 0.05, ^★★^*P* < 0.01 versus control).

Due to the significantly activated iron-starvation stress, we, therefore, raise the hypothesis that the synergistic cytotoxicity of DHA/DDP may result from free iron accumulation. Firstly, iron chelator DFO was utilized to verify this hypothesis that DFO inhibited the synergistic cytotoxicity of DHA/DDP-induced ferroptotic cell death, as evidenced by the following: (i) DFO mitigated DHA/DDP-induced ferroptosis in a concentration-dependent manner (Fig. 6A), (ii) Fe^2+^ addition accelerated DHA/DDP-induced ferroptotic cell death which was able to be blocked by DFO treatment (Fig. 6B), and (iii) DFO also exhibited a strong effect on the alleviation of free iron accumulation and cellular ROS peroxidation (Fig. 6C, D). Moreover, we confirmed that DFO could ameliorate the mitochondrial morphogenesis change as effectively as mitoQ (Fig. 6E). Notably, DFO attenuated the mitochondrial dysfunction resulting from DHA/DDP treatment and rescued the mitochondrial respiration and ATP production (Fig. 6F, G), as evidence by the detection of oxygen consumption. These data indicated that free iron accumulation resulting from DHA/DDP treatment acts upstream of mitochondrial dysfunction. Lastly, the production of lipid peroxides was monitored by the staining of Bodipy C11 probe, images showed that DFO ameliorated the production of lipid ROS, as effective as Fer-1 (Fig. 6H, I). Collectively, these data indicate that pharmacological chelation free iron rescues DHA/DDP-mediated ferroptotic cell death and mitochondrial dysfunction.

### Reconstituted expression of FTH contributes to the tolerance of DHA/DDP-induced ferroptosis

**Fig. 7:**
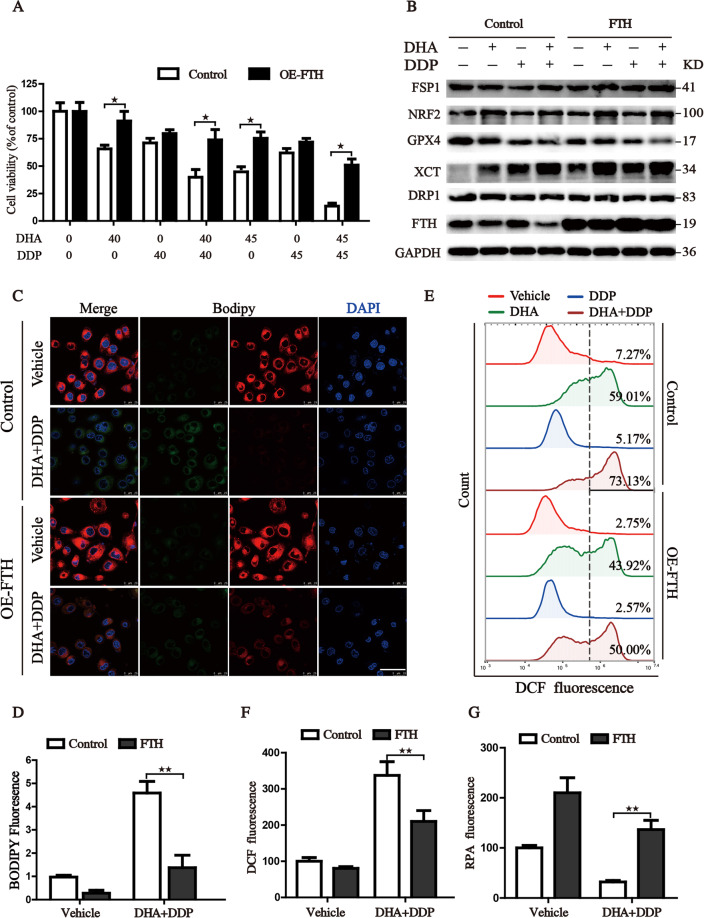
Reconstituted expression of FTH contributes to the tolerance of DHA/DDP-induced ferroptosis. **A** PANC1 cells were treated with various concentrations of DHA and DDP for 24 h, followed by CCK8 assay. **B** Proteins isolated from the treated cells were assayed by western blot to detect the expression of ferroptosis-related proteins. **C**, **D** Indicated cells were stained with BODIPY C11 probe for the assessment of lipid ROS production and observed under the confocal laser microscope. Scale bars: 50 μm. Cells with indicated treatment were stained with RPA and DCF-DA probe for 30 min and followed by flow cytometry to assess intracellular Fe^2+^ and ROS level (**E**–**G**) (values represented mean ± SD. ^★^*P* < 0.05, ^★★^*P* < 0.01 versus control).

Ferritin heavy chain (FTH) not only harbors ferroxidase activity which could catalytically oxidize Fe^2+^ to inactive Fe^3+^, but also plays a vital role in maintaining iron homeostasis by storing iron in a soluble, non-toxic form for its detoxification. Previously, studies have shown that free iron released from lysosomal degradation of ferritin plays a critical role in ferroptosis [21, 23, 24]. Given that synergistic cytotoxicity of DHA/DDP resulted from FTH degradation, a process that gives rise to the free iron accumulation. We then discovered whether genetically enforced expression of FTH could abolish the synergistic cytotoxicity of DHA/DDP. Firstly, the FTH enforced expression PANC1 cells were constructed and transfection efficiency was verified by western blot (Fig. S3). Then, the FTH-PANC1 cells were subjected to different concentrations of DHA and/or DDP. Cell viability assay indicated that FTH efficiently relieved the cytotoxicity of DHA/DDP (Fig. 7A). Furthermore, the results from western blot assay manifested that this cytoprotection effect mainly originates from the lesser degradation of FTH, but not from modulating other ferroptosis regulators, such as GPX4 or XCT (Fig. 7B). FTH reconstituted PANC1 cells also exhibited decreased intracellular labile iron pool, reduced cellular ROS lever and diminished lipid peroxides generation when co-treated with DHA and DDP (Fig. 7C–G). Accordingly, we come to a conclusion that reconstituted expression of FTH is able to abolish synthetic cytotoxicity of DHA/DDP through chelating intracellular labile iron pool.

### The combination of DHA/DDP treatment inhibits the growth of xenografts in vivo

**Fig. 8:**
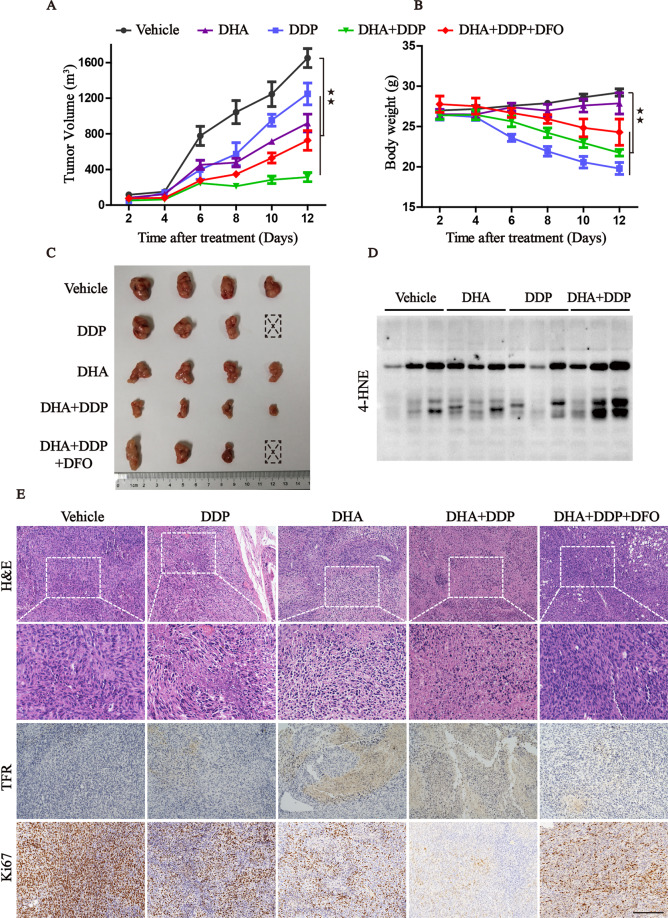
The combination of DHA and DDP inhibits the growth of xenograft in vivo. **A**, **B** Xenografts were established in mice and treated with vehicle, DHA, DDP, DHA/DDP, and DHA/DDP plus DFO, respectively. The tumor volume and body weight were measured every 2 days. **C** The dissected xenografts were photographed at the end of the experiment. **D** Western blots analysis for 4-HNE on lysates isolated from xenografts in different groups. **E** H&E staining and IHC analysis for TFR, Ki67 in indicated tumor specimens. Scale bars: 200 μm (values represented as mean ± SD. ^★★^*P* < 0.01 versus control).

In order to study the in vivo therapeutic potential of DHA/DDP combination, we established the subcutaneous tumor model bearing PANC1 cells and randomly divided them into five groups with indicated treatment. Mirroring our in vitro data, the in vivo data further demonstrated that administration of DHA or DDP suppressed tumor growth and the antitumor efficacy was more pronounced in DHA/DDP combination-treated group compared to DDP or DHA mono-treated group (Fig. 8A, C). Modulating iron homeostasis through pharmacological treatment of iron chelator DFO could weaken the effect of DHA/DDP in vivo. It is well known that DDP exhibits severe side-effects, we also focus on animal weight as an indicator of drug toxicity. As shown in Fig. 8B, DDP-treated animals exhibited a significant loss of weight, while administration of DHA did not exhibit obvious toxicity with little loss of mice’s body weight. Unexpectedly, DDP co-treatment with DHA not only reduced the tumor growth but also partly alleviated the toxicity of DDP, suggesting higher safety for clinical use. The immunoblotting assay of lysates from tumor tissues also verified the in vitro observations that DHA co-administration with DDP synergistically induced lipid peroxidation locally, with the dramatic increasing of 4HNE (Fig. 8D). In addition, the IHC assay displayed that DHA acted synergistically with DDP to increase the area of necrosis, augment the expression of TFR, and decrease the Ki67 staining. Whereas, administration of DFO could significantly reverse these phenotypes by suppressing ferroptosis (Fig. 8E). Our findings show that the combination of DHA with DDP is not only tolerable but also beneficial, which may act as a promising therapeutic agent for treating PDAC.

## Discussion

Distinct lethal subroutines are designed for selectively targeting cancer cells via diverse regulated cell death (RCD) processes, including apoptosis, ferroptosis, pyroptosis, necroptosis, etc. Each of the RCD processes is modulated by unique signal transduction pathways and differentially affect tumor response to treatment. The most extensively studied type of RCD is apoptosis, whose activation relies on the cleavage of intracellular proteases’ caspase. However, the clinical application of the therapeutic approach through inducing apoptosis in oncology remains an insurmountable challenge for the high resistance rate [25]. Thus, targeting non-apoptotic RCD processes may provide an alternative strategy for suppressing tumor growth.

Ferroptosis is a non-apoptotic, RCD first observed in cancer cells with oncogenic *Ras* mutation in 2012 [26], which is characterized as a catastrophic accumulation of free iron and unrestricted lipid peroxidation. Several signal transduction pathways including iron metabolism, GSH-GPX4, and FSP1-COQ10 constitute the core molecular mechanism of ferroptosis. Increasing evidence has revealed that inducing ferroptosis could act as a promising therapeutic strategy and eliminate the resistance of cancer cells to drug-induced apoptosis [27]. The mutated *RAS* family genes are the most common oncogenes in pancreatic cancer that could be selectively targeted by erastin through inducing ferroptosis [28, 29]. Activation of the RAS/RAF-MEK/ERK signaling pathway is required for erastin-induced cell death, while restraining *RAS* or its downstream signaling molecules reverses the lethality against cancer cells [30, 31].

Previous studies have demonstrated that DHA and cisplatin exert synergistic anti-angiogenic and anti-tumor effects through inducing apoptosis [32, 33]. In this study, we found DHA could effectively optimize its antitumor activity of DDP, and significantly reduced its effective concentrations. In addition, combination of DHA and DDP dramatically impaired mitochondrial homeostasis and suppressed the proliferation of pancreatic cancer cells. Further experiments revealed that the cell death could be blocked by ferroptosis inhibitor, iron chelator, and lipid ROS scavenger, rather than apoptosis or necroptosis inhibitors. Importantly, accumulation of lipid peroxides detected by fluorescence staining of MDA, 4-HNE, or BODIPY C11 was observed prior to the onset of cell death, which could be rescued by pharmacological chelation free iron or genetically enforced expression of FTH. These results, consistent with a previous study, manifested that it is a promising strategy to induce ferroptosis in PDAC [34]. Collectively, our data together with other preclinical findings support the notion that induction of ferroptosis might constitute a suitable strategy against PDAC tumors.

Iron is an essential micronutrient and enables the function of vital enzymes [35]. Available amount of free iron is essential for the process of electron transport, cellular respiration, cell proliferation and differentiation, and gene expression regulation in cancer cells. However, overload of labile iron pool is biochemically dangerous with the high capacity to promote the formation of ROS via the Fenton reaction, leading to severe damage to the main cellular biomolecules. As reported, free iron levels can be used as a criterion to determine which type of cancer is more prone to benefit from ferroptosis-promoting therapies [36]. Iron-rich tumors (such as PDAC [37], HCC [38], breast cancer [39], and NSCLC [40]) could be more suitable to therapies of inducing ferroptosis, and more sensitive to agents that promote ferroptosis. Additionally, subsequent studies have identified that mutant *RAS* signaling enriches the cellular iron reservoir via transcriptional regulation of iron metabolism genes [30]. In the present study, we have provided evidence to support a means to induce ferroptosis in PDAC via modulation of iron metabolism. First, DDP combined with DHA significantly accelerated the accumulation of labile free iron reflected by the decreased fluorescence of RPA, and increased expression of TFR, which subsequently results in lipid peroxidation. Second, DFO mitigated DHA/DDP-induced ferroptosis in a concentration-dependent manner, while Fe^2+^ addition accelerated DHA/DDP-induced ferroptotic cell death which was able to be blocked by DFO treatment. Third, reconstituted expression of FTH is able to abolish synergistic cytotoxicity of DHA/DDP through chelation intracellular transitional iron pool. Lastly, pharmacological treatment of iron chelator DFO could weaken the effect of DHA/DDP on ferroptosis in vivo. Our data show that the combination of DHA with DDP is not only tolerable but could also be beneficial through inducing ferroptosis.

DDP is still used as a mainstay of chemotherapeutic agent in combination with other drugs or radiotherapy for pancreatic cancer therapy [41]. However, DDP is commonly associated with acquired drug resistance and high toxicity in clinical settings. Previous research regards that DDP leads to DNA injury and ultimately induces apoptosis [42]. Recent studies have discovered that DDP could induce GSH depletion and GPX4 inactivation, which emerges as an inducer of ferroptosis [12, 43]. Similarly, DDP-resistant or platinum-tolerant cancer cells were also shown to exhibit increased vulnerability to ferroptosis [44]. In our present work, we observed that DDP acts synergistically with DHA to induce oxidative stress which originated from impaired mitochondrial homeostasis. Mechanically, DHA acts synergistically with DDP to suppress mitochondrial oxidative phosphorylation process through inhibiting basal respiratory, maximal respiratory, non-mitochondrial oxygen consumption, thus leading to the reduction of ATP production and enhancement of mitochondrial ROS generation. Iron depletion rescued the mitochondrial respiration and ATP production under the challenge of DHA/DDP, which indicated that free iron accumulation originating from DHA/DDP treatment acts upstream of mitochondrial dysfunction. In addition, we found that co-treatment of DHA with DDP synergistically decreased the expression of GPX4. This may be another mechanism involved in the activation of ferroptotic cell death.

Furthermore, several completed clinical trials (NCT00764036, NCT02353026) and ongoing clinical trials (NCT02633098 and NCT03093129) have shown the efficiency and great tolerance of artemisinins in patients with solid tumors. In addition to inducing apoptosis, both DHA and DDP can trigger ferroptosis in cancer cells and exhibit the synergistic effect through increasing the intracellular free iron. Therefore, our study provides evidence that DHA may be a promising adjuvant to improve the cisplatin-based treatment of patients with pancreatic cancer. We also provide a framework for further understanding and targeting of ferroptosis in cancer therapy.

## Conclusion

This study presents strong evidence that DHA could intensively strengthen the cytotoxicity effect of DDP and significantly reduce its effective concentrations both in vitro and in vivo. In particular, ferroptosis contributes to the main cytotoxic effects in PDAC cells under the challenge of DHA and DDP. Our results provide experimental evidence that DHA acts synergistically with DDP and renders PDAC cells vulnerable to ferroptosis, which may act as a promising therapeutic strategy.

## Supplementary information

Figure S1.

Figure S2.

Figure S3.

## Data Availability

All data generated during this study are included either in this article or in the supplementary information files.
