# Supplementary figures and images for: DHA exhibits synergistic therapeutic efficacy with cisplatin to induce ferroptosis in pancreatic ductal adenocarcinoma via modulation of iron metabolism
Source: Cell Death Dis. 2021 Jul 15;12(7):705. doi: 10.1038/s41419-021-03996-y (PMC8280115; doi:10.1038/s41419-021-03996-y)

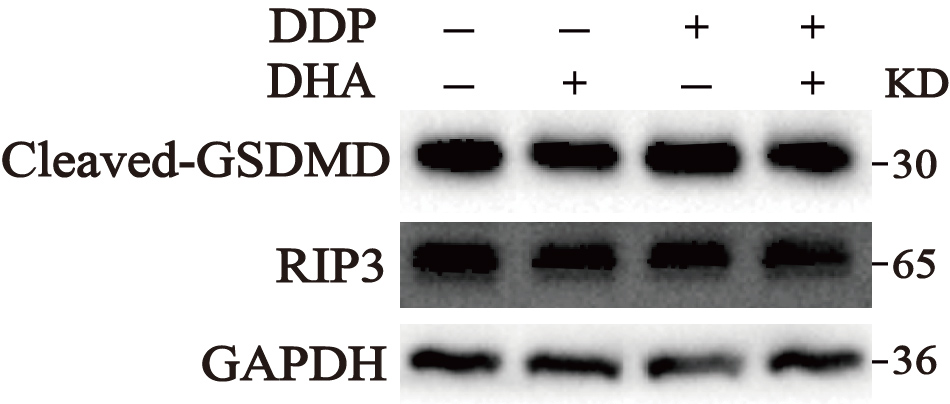

Supplement: Supplementary file 1 — Figure S1. [file 41419_2021_3996_MOESM1_ESM.jpg]

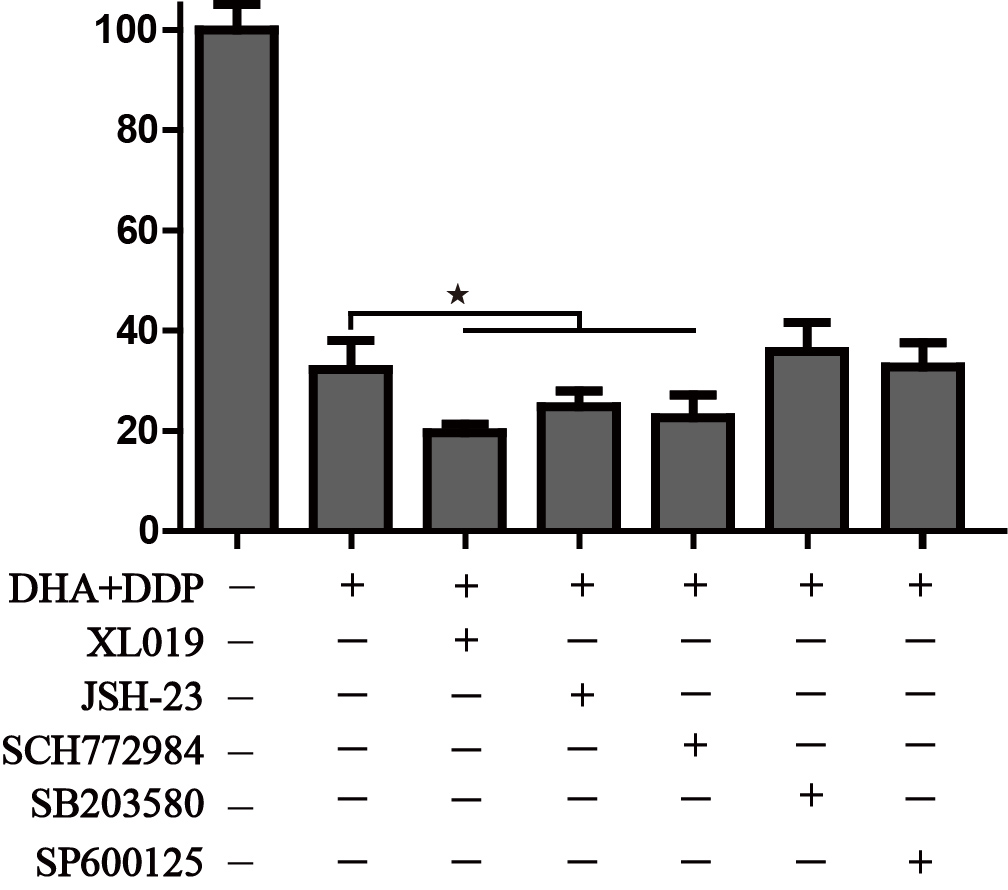

Supplement: Supplementary file 2 — Figure S2. [file 41419_2021_3996_MOESM2_ESM.jpg]

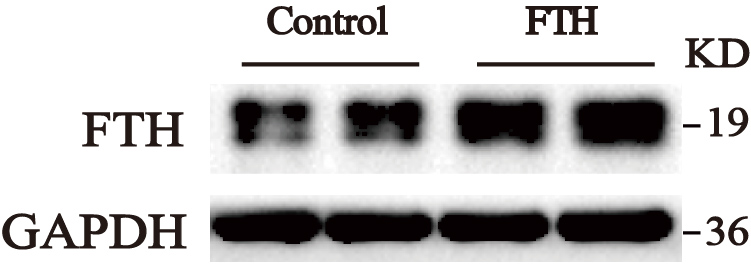

Supplement: Supplementary file 3 — Figure S3. [file 41419_2021_3996_MOESM3_ESM.jpg]
